# RP1 Is a Phosphorylation Target of CK2 and Is Involved in Cell Adhesion

**DOI:** 10.1371/journal.pone.0067595

**Published:** 2013-07-03

**Authors:** Frank Stenner, Heike Liewen, Stephan Göttig, Reinhard Henschler, Norbert Markuly, Sascha Kleber, Michael Faust, Axel Mischo, Stefan Bauer, Martin Zweifel, Alexander Knuth, Christoph Renner, Andreas Wadle

**Affiliations:** 1 Division of Oncology, University Hospital Zurich, Zurich, Switzerland; 2 DRK Institute of Transfusion Medicine and Immune Hematology, Johann-Wolfgang Goethe University, Frankfurt am Main, Germany; 3 Institute of Medical Biochemistry and Molecular Biology, University of the Saarland, Homburg/Saar, Germany; 4 Medical Oncology Office, Lebach, Germany; 5 Institute of Medical Oncology, Inselspital, Bern, Switzerland; Seoul National University, Republic of Korea

## Abstract

RP1 (synonym: MAPRE2, EB2) is a member of the microtubule binding EB1 protein family, which interacts with APC, a key regulatory molecule in the Wnt signalling pathway. While the other EB1 proteins are well characterized the cellular function and regulation of RP1 remain speculative to date. However, recently RP1 has been implicated in pancreatic cancerogenesis. CK2 is a pleiotropic kinase involved in adhesion, proliferation and anti-apoptosis. Overexpression of protein kinase CK2 is a hallmark of many cancers and supports the malignant phenotype of tumor cells. In this study we investigate the interaction of protein kinase CK2 with RP1 and demonstrate that CK2 phosphorylates RP1 at Ser^236^ in vitro. Stable RP1 expression in cell lines leads to a significant cleavage and down-regulation of N-cadherin and impaired adhesion. Cells expressing a Phospho-mimicking point mutant RP1-ASP^236^ show a marked decrease of adhesion to endothelial cells under shear stress. Inversely, we found that the cells under shear stress downregulate endogenous RP1, most likely to improve cellular adhesion. Accordingly, when RP1 expression is suppressed by shRNA, cells lacking RP1 display significantly increased cell adherence to surfaces. In summary, RP1 phosphorylation at Ser^236^ by CK2 seems to play a significant role in cell adhesion and might initiate new insights in the CK2 and EB1 family protein association.

## Introduction

The EB1 family proteins encoded by three distinct genes (MAPRE1–3) are involved in microtubule stability and integrity [1], [2]. Since their discovery from 1995 to 2001, various cellular functions of these proteins have been reported [3]–[5]. The common functional motif of EB1, RP1 and EB3 proteins is binding to microtubules, but further divergent individual functions seem to exist. For EB1, the best studied member of the family a [1], [5]. In humans, EB1 was detected as an adenomatous polyposis coli (APC)–interacting protein whose binding domain was affected by APC mutations implicated in colon cancer [3]. APC by itself is a key regulator within the unit pathway. APC, as part of a degradation complex, down-regulates intracellular β-catenin hereby disrupting signaling of this pathway [6], [7]. EB3 (EBF3) a close homolog of EB1 is preferentially expressed in brain tissue [8] binds to APC and has been implicated in MT bundling [1].

Until now little functional information is available for the second EB1 family member RP1. Regulatory mechanisms governing its cellular function are hitherto unknown. Post transcriptional expression control of RP1 by a viral MicroRNA (miR-US25-1 from human cytomegalovirus, CMV) has been described [9] but no endogenous mammalian micro RNAs for RP1 have been discovered yet.

Recently, RP1 has been identified in a proteomic screen of pancreatic cell lines that had specifically been selected for increased perineural invasiveness [10]. In that study high expression of MAPRE2 (RP1) mRNA was associated with poor outcome and prognosis.

With a comparable molecular weight of 30–37 kDa and a size of 268–327 amino acids, the overall homology among the EB1 protein family members averages between 70% and 77% identity and is specifically higher at their C- and N-terminus. All three family members share a N-terminal calponin-like or actin-binding domain and an EB1-like C-terminal domain. Within these domains conservation is high reaching over 90% in the N-terminal and above 80% in the C-terminal domain, respectively. Dimerization of the EB1 proteins depends on their C-terminal moieties. All EB proteins homodimerize, but only EB1 and EB3 have the ability to heterodimerize [11].

The interjacent region of the two homologous domains shows the greatest variability between the three proteins. Within this distinctive zone a serine rich stretch is notable only in the RP1 protein sequence. Using different prediction algorithms, we identified a potential CK2 phosphorylation site that is not present in EB1 or EB3. The serine residue at position 236 displays a classical CK2 (X-S/T-X-X-D) motif and is conserved among various species with an inconsequential variety in the X-residues.

CK2 is a pleiotrophic ubiquitous and constitutively active protein kinase with a broad range of targets [12]. Though not an oncogene itself, CK2 supports cancer cells by delivering proliferative signals and protection from apoptosis (for review see [13] and [14]). Finally, CK2 has been implicated in cell adhesion by its phosphorylation of Vitronectin [15].

This study examines the relationship between CK2 and RP1 and a putative role of RP1 phosphorylation in adhesion.

## Materials and Methods

### Reagents and Antibodies

The antibodies against RP1 and their usage have been described elsewhere [4], [16]. A monoclonal mouse antibody against α-tubulin (Sigma-Aldrich, St. Louis, USA) was used in a dilution of 1:10000, for Western blotting. The C-terminal antibody against N-cadherin (clone C32, from BD Transduction Labs, NJ, USA) was applied 1:3000 for Western blotting and 1:300 for FACS and immunofluorescence (IF). The N-terminal antibody against N-cadherin (Cell Signalling) was employed 1:1000 for Western blotting and 1∶100 for FACS and IF.

Phalloidin-Rhodamine (Molecular Probes) was used at a final concentration of 0.17 µM. DNAse I (Invitrogen desoxyribonuclease I, Alexa Fluor 488 conjugate) was diluted with PBS to a working concentration of 40 µg/ml. The Actin FACS analysis was carried out as described by Knowles and McCulloch [17].

Horseradish peroxidase conjugated secondary antibody goat anti mouse (BioRad®, Munich, Germany), goat anti rabbit (Sigma-Aldrich, Munich, Germany) and donkey anti goat (Millipore Corporation, Billerica, USA) were used diluted 1∶3000 for Western blotting.

The conjugated secondary antibody goat-anti-mouse FITC (Invitrogen, Basel, Switzerland) was employed 1∶500 both in FACS and IF and the goat-anti-rabbit FITC (BD Transduction Labs) 1∶800 for FACS.

The antibodies against CK2 subunits (α-CK2/α-CK2α/α-CK2β) used in this study were characterized and described by Faust *et al.* [18]. For all Western blots these CK2 antibodies were diluted 1∶2000, for interaction assays 1∶50. In addition, anti-RP1 (polyclonal antibody from Santa Cruz) was used for Western blotting experiments in a dilution of 1∶300.

### Plasmids

The RP1 wild type (RP1-wt) DNA sequence, initially described by Juwana *et al.* [16], was subcloned into the pEAK8 vector (Edge BioSystems, Gaithersburg, MD, US) utilizingg the *EcoR* I and *Not* I restriction sites. In this vector RP1 gained a c-terminal 6xHis-Tag.

From the new master clone site-directed mutagenesis of various RP1 constructs concerning the Ser^236^ residue were performed by using the QuikChange II site-directed mutagenesis kit (Stratagene, La Jolla, CA), according to the manufacturer's instructions. The following primers were used:

RP1-ALA-forward: 5′-GTGGCTCAGCATCCAAGGCAGATAAAGATTTAG-3′

RP1-ALA reverse: 5′-CTAAATCTTTATCTGCCTTGGATGCTGAGCCAC-3′

RP1-ASP-forward: 5′-GTGGCTCAGCATCCAAGGATGATAAAGATTTAG-3′

RP1-ASP-reverse: 5′-CTAAATCTTTATCATCCTTGGATGCTGAGCCAC-3′

The mutations were then confirmed by DNA sequence analysis (GATC, Konstanz, Germany).

### Cells Lines

Human embryonic kidney cells (HEK293) were obtained from the American Type Culture Collection (Manassas, VA, USA). For the radiolabelling experiments HEK293 cells were cultured in DMEM without sodium pyruvate and phosphate. For generation of stable cell lines HEK293 cell lines were transfected using LipofectAMINE Plus (Invitrogen) with the respective RP1 encoding plasmids or empty vector.

Clones outgrowing under puromycine pressure were tested by Western blotting for protein expression levels and by immunofluorescence. To avoid clonal bias three different clones from each construct were pooled and propagated in culture for at least four weeks before the respective experiments.

### CK2/RP1 Interaction Assay (Co-immunoprecipitation)

**Figure 1:**
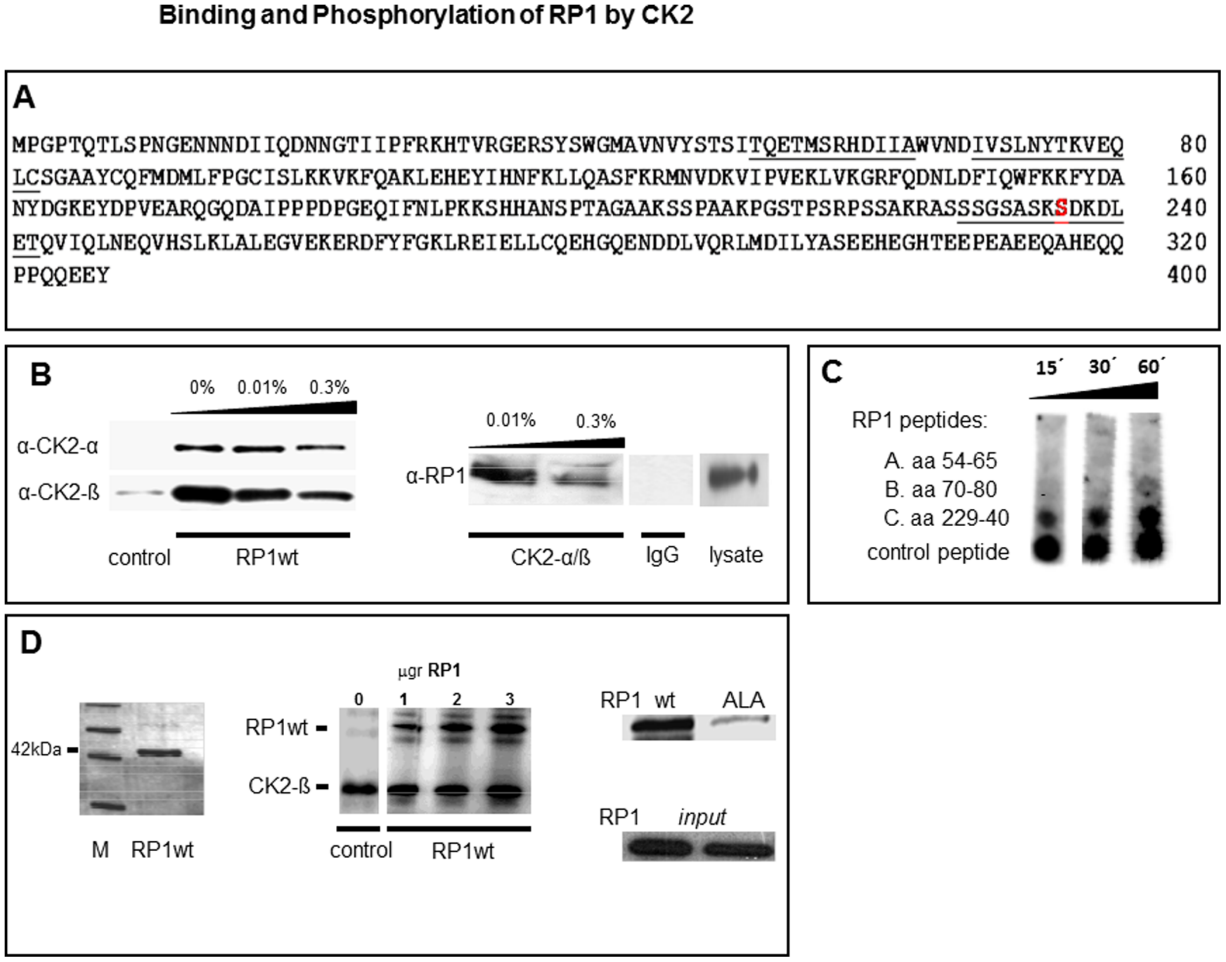
Binding and Phosphorylation of RP1 by CK2. **1A** Identification of CK2 phosphorylation site - RP1-sequence (amino acid), three potential CK2 kinase sites S59, S72, S236 (underlined) were identified by prosite scan (www.expasy.ch). The peptides used for in vitro experiments (1C) are marked in bold. S236 the actual CK2 phosphorylation site is shown in red. **1B** Interaction–assay RP1/CK2 - Endogenous RP1 (first panel) was co-precipitated with its potential binding partners. RP1/CK2 kinase interaction could be detected by specific α/ß CK2 subunit antibodies. The black wedges in this panel indicate increasing stringency of washing procedure (% Tween20/PBS). In a reverse experiment (right side panel), endogenous RP1 was verified as genuine CK2 binding substrate. By using CK2 subunits as baits, RP1 could be detected in the pulldowns by its specific RP1 antibody (right panel). No signal was seen when an insignificant IgG antibody was used. On the far right 1/10 of cell lysate of the foregoing experiments is depicted as an input control. The black wedges in this panel indicate stringency of the washing procedure (0.01% and 0.3% Tween/PBS). **1C** Biotinylated peptides (A: aa54–65, B: aa70–80, C: aa229–240) containing the potential CK2 phosphorylation sites S*^59^*, S*^72^*, S*^236^ were* synthesized and tested as CK2 phosphorylation substrates (A, B, C, 3 µg each) in an *in vitro* phosphorylation assay. A known positive CK2 kinase site peptide (DDDDSDDDDD, 3 µg) served as a control. The black wedge indicates incubation times (minutes). **1D** CK2 kinase assay - Recombinant CK2 and ^33^P-gamma-ATP were incubated in vitro with different amounts of RP1-wt protein (first panel shows a coomassie stain of his-tagged purified RP1 protein used for the assay) and phosphorylation was measured by autoradiography (middle panel). The amounts of RP1 protein used are indicated above the middle panel. Autophosphorylation of CK2 at its subunit ß served as positive control RP1-ALA236 mutated protein (ALA) was almost non-phosphorylated in comparison to the wild type protein (right side upper panel). The lower panel on the right side shows a coomassie stain representing the amount of RP1 used for this experiment.

His-tagged RP1 wild type protein and mutants were isolated with Ni-NTA-agarose beads from lysates of HEK293 cells stably expressing the respective RP1 construct. After stringent washing with increasing amount of PBS/Tween (0%–0.3%) lysates were run on 10% SDS PAGE gels and subsequently blotted to nitrocellulose membranes. These membranes were probed with CK2 subunit antibodies as indicated in Fig. 1C. Lysates from cells bearing the corresponding empty vector were used as controls. For the reverse experiment CK2 antibodies were used for precipitation and the membrane was probed with respective RP1 antibody detecting endogenous RP1.

### Phosphorimaging

For analysis, the protein gels were stained by either Pro Q Diamond (Molecular Probes, Eugene, OR, USA) or by Sypro Ruby (Biorad, Munich, Germany) according to the manufactures’ protocols. Phosphorimaging of stained gels was performed by using either a Storm 860 (at 450 nm) or a Typhoon model 9410 (at 512 nm with 560 V) (Amersham Pharmacia Biotech, Freiburg, Germany) and ImageQuant Version 5.1 software (Amersham).

### Kinase Assays

Potential CK2 kinase sites S*^59^*, S*^72^*, S*^236^* were identified by the software program Prosite located at www.expasy.ch/prosite and then synthesized as peptides (at the Institute of Biochemistry, University Saarland, Homburg/Saar, Germany). Three µg of these peptides and an irrelevant control peptide were then tested as CK2 phosphorylation substrates using a ^33^P based phosphorylation assay described before [19]. The resulting signals were scanned with a phosphorimaging device as described before. For CK2 kinase assays involving wild type RP1, His-tagged RP1 protein was isolated by Ni-NTA-agarose beads from lysate of HEK293 cells stably expressing corresponding plasmid DNA. His-tagged RP1, RP1-ALA^236^ and RP1-ASP^236^ (0.5–1 µg) were incubated with recombinant CK2 and kinase buffer (50 mM Tris-HCL pH 7.5,150 mM NaCl, 5 mM MgCl_2_,1 mM DTT) containing 2 µCi ^33^P-gamma-ATP, for 30 minutes at 37°C. The bead/protein complex was dissolved by 100 mM imidazol and centrifuged. The supernatant was subjected to SDS-PAGE and subsequently blotted to nitrocellulose membranes. For control purposes CK2 was incubated with 2 µCi ^33^P-gamma-ATP and kinase buffer only. The membranes were examined by phosphorimaging.

### FACS Assays

For flow cytometric analysis cells were seeded into T-25 flasks at 10^4^ cells/cm^2^. On day 3 cultured cells (5×10^5^) were fixed as described below and stained with the respective antibodies. Primary antibodies and secondary antibodies were used as described in the section antibodies above. By FACS (FACSCalibur, Becton Dickinson) 1×10^5^ cells were subjected to analysis in each individual experiment.

For measurement of N-cadherin level cells were pretreated with a fixation/intracellular staining buffer Cytofix/Cytoperm Kit (BD Biosciences) and incubated for 30 minutes with a monoclonal antibody directed against the cytoplasmic tail (clone C32, from BD Transduction Labs, NJ, USA) on ice. After one wash with PBS the corresponding secondary antibody goat-α-mouse FITC (Invitrogen, Basel, Switzerland) was applied for 15 minutes immediately followed by FACS analysis. For negative controls of the FACS experiments primary antibodies were omitted and cells incubated only with a secondary antibody. Complementary cells from the above experiments were mounted onto slides by cytospin and examined by immunofluorescence with a 40× magnification (data not shown).

### Determination of G and F Actin Content in RP1 Expressing Cells

The G- and F-actin content of constitutively RP1 expressing cells were measured following the method described by Knowles and McMulloch [17]. The experiments were done in triplicate and showed distinct staining patterns of G-actin and F-actin with very little fluorescence crosstalk.

### Adhesion of RP1 Mutants on Endothelial Cells Under Flow

Parallel plate flow chambers (µ-slide, Ibidi, Martinsried, Germany) were seeded with human umbilical vein endothelial cells (HUVECs, Cambrex Bio Science, Verviers, Belgium). The cells were grown in EGM-2 medium (BulletKit, Cambrex Bio Science, Cambridge, UK) until reaching confluency. Prior to experiments, HUVECs were stimulated overnight with 10 ng/mL TNF alpha. HEK293 cells stably transfected with RP1 isoforms (RP1-wt, RP1-ALA^236^, RP1-ASP^236^ or the empty vector bearing cell line as a control) were grown until reaching cell densities of 75%. Then, cells were trypsinized, washed and resuspended in HBSS/0.1% BSA. 1×10^5^ HEK293 cells were added to the flow chamber and allowed to settle for 3 min as described before [20]. After initial adherence, shear stress of 0.2 dyn/cm^2^ was applied by flushing 37°C prewarmed HBSS/0.1% BSA using a precision pump (IPC, Ismatec, Wertheim-Mondfeld, Germany) until no cells were further released. Subsequently, shear stress was increased stepwise every 30 seconds to a maximum of 2.5 dyn/cm^2^. After each step, cells were photographed using a CCD camera (Sony, Cologne, Germany) mounted on an inverted stage microscope (Axiovert 135, Zeiss, Oberkochen, Germany). Adherent cells were counted manually in four representative fields for every shear stress condition and values were normalized to the number of initially adherent cells set at 100% [20].

### Shear Stress and Endogenous RP1 Expression

Fluid shear stress experiments were performed as described by dela Paz and colleagues [21]. In brief, HEK293 cells were grown in the periphery of 100-mm culture dishes through blocking the center of the dish by a 60-mm culture dish (TPP Techno Plastic Products AG, Trasadingen, Switzerland) until reaching cell densities of 70%. Then shear stress of 1 dynes/cm^2^ was applied while shaking cells at 46 rpm with an orbital rotation of 1.1 cm inside a cell incubator approximating the shear stress across each cell layer as the maximal wall shear stress: τmax = a√ρη(2πf)^3^, where a is the radius of orbital rotation (1.1 cm), ρ is the density of the medium (1.0 g/ml), η is the viscosity of the medium (7.5×10^−3^ dynes·s/cm^2^) and f is the frequency of rotation (rotations/second). As a control HEK293 cells were incubated only. After five hours cells were lysed and 1mg of protein per treatment group was used for immunoprecipitation following the manurfacturer’s protocol (Pierce Crosslink IP Kit, Rockford, USA). 15 µg goat anti RP1 antibody (Santa Cruz Biotechnology) was bound to agarose and incubated with each cell lysate. Bound proteins were then eluted from the complex, run on a 10% SDS-Page gel, transferred to nitrocellulose membrane with a pore size of 0.22 µm. The membrane was blocked with Roti-Block (Carl Roth GmbH&Co.KG, Karlsruhe, Germany) for 2 hrs and incubated with goat anti RP1 antibody. Bound antibody was detected applying peroxidase conjugated donkey anti goat IgG and TMB (GE Lifescience) according to manufacturer’s protocol. As loading control 20 µg of lysate protein from HEK293 (sheared and non sheared) cells was analyzed in a Western blot procedure employing mouse anti α-tubulin antibody. Western blot signals were quantified exerting ImageJ software (Rasband, W.S., ImageJ, U. S. National Institutes of Health, Bethesda, Maryland, USA).

### Phosphorylation of RP1 Under Shear Stress

HEK293 RP1-wt cells were grown and exposed to shear stress of 1 dynes/cm^2^ for five hours as described above. Thereafter, cells were lysed and 1mg of protein was used for immunoprecipitation. 10 µg monoclonal mouse anti-His antibody (Genscript, Piscataway, USA) were linked to agarose and used as probe on the cell lysate. Bound proteins were eluted, run on a 10% SDS-Page gel and transferred to nitrocellulose membrane. The blocked membrane was incubated with 2 µg/ml rabbit anti-Phosphoserine Antibody (Invitrogen Corporation, Camarillo, USA). Bound antibody was detected applying peroxidase conjugated goat anti-rabbit IgG and TMB. As loading control lysates from HEK293 RP1-wt (sheared and non-sheared) cells were detected in a Western blot procedure employing goat anti-RP1 antibody.

### Adherence Under Shear Stress

HEK293 cells stably expressing RP1-ALA^236^, RP1-ASP^236^, empty vector, shRNA or sh-control were grown until reaching cell densities of 70%, detached and 2×10^6^ cells per cell line were seeded in the periphery of 100-mm culture dishes and incubated one hour until initial adherence. Thereupon, shear stress of either 1.5, 4.5 or 8.5 dynes/cm^2^ by shaking the cells at 60 rpm, 125 rpm or 190 rpm was applied for 5 min. Medium and floating cells were removed, cells were stained with crystal violet solution (0.1%) for 25 min at room temperature and rinsed 15 min in ddH_2_O. Plates were dried on paper and crystal violet dissolved from cells by 0,5% Triton X-100 while shaking for one hour and measured at 570 nm on an Epoch 96-well plate reader (BioTek Instruments,Winooski, USA).

### Generation of shRNA Cell Lines

10^5^ HEK293 cells per well were seeded in a 12-well-plate and cultivated overnight in a cell incubator (at 37°C and 5% CO2). The next day cells were treated with 4 µg/ml Polybrene (Santa Cruz Biotechnology) for 2 hrs followed by 0.1×10^6^ lentiviral particles of RP1 shRNA (Santa Cruz Biotechnology) or control shRNA lentiviral particles. After 48hrs cells were transferred to a cell culture flask and 24 hrs later growth medium was supplemented with 5 µg/ml Puromycin dihydrochloride (Santa Cruz Biotechnology). Single cells were seeded in 96 well plates, grown and 10 µg of protein lysates from different colonies were run on an 10% SDS page gel. Protein was transferred to a nitrocellulose membrane, which was cut horizontally at a protein marker size of 45 kDa. For RP1 detection the lower membrane part was exposed to goat anti-RP1 antibody. For loading control purposes the upper membrane part was incubated with mouse anti α-tubulin antibody (Fig. S3).

### Statistics

Statistical tests were performed using the GraphPad Prism version 5.03. (www.graphpad.com). For p value calculation the two-tailed Fisher′s exact test was employed unless otherwise noted.

## Results

In a yeast-two-hybrid-assay CK2 kinase had appeared as a potential interaction partner of RP1 (own unpublished data). In this study we have identified three potential CK2 phosphorylation site motifs (X-S/T-X-X-D) at amino acid position 59, 72, 236 within the RP1 sequence (Fig. 1A). Whereas the potential CK2 phosphorylation site motif at position 52 is present in all EB1 family members, the phosphorylation sites 72 and especially 236 are not found in the two other EB1 family members EB1 and EB3 (despite a sequence identity of >70% among all family members including RP1). To test whether CK2 interacts with RP1, we performed co-precipitation experiments in RP1-expressing HEK293 cells. CK2 was recovered in RP1 immunoprecipitates using different CK2 antibodies (Fig. 1B, left panel). In contrast, control immunoprecipitates from cells not expressing RP1, did not or barely contain CK2. In the reverse experiment, CK2 immunoprecipitates contained RP1 (Fig. 1B, right panel). These results indicate that RP1 and CK2 interact *in vivo*.

To examine which of the three identified phosphorylation sites are actually phosphorylated by CK2 *in vitro* kinase assays were performed. Synthesized peptides of RP1 containing S59, S72 and S236 were used as phosphorylation targets. Only the S236 containing peptide and a positive control (DDDDSDDDDD) were phosphorylated by CK2, whereas peptides S59 and S72 were not or only marginally phosphorylated (Fig. 1C). Having shown the importance of serine at position 236 we replaced this amino acid by alanine or aspartate in an RP1-containing plasmid. These RP1 mutants, a non-phosphorylatable RP1-ALA^236^ (ALA) and a Phospho-mimicking RP1-ASP^236^ (ASP) were used in a kinase assay and incubated with recombinant CK2. Wild type RP1 was considerably phosphorylated, whereas RP1-ALA^236^ was significantly less phosphorylated (Fig. 1D). The weak band seen in RP1-ALA^236^ lane (Fig. 1D upper right lane, right panel) is most likely a less specific phosphorylation at a different serine site, e.g. S72. Together, the *in vitro* kinase assays showed that CK2 phosphorylates RP1 predominantly at serine 236.

Furthermore, phenotypic changes of the mutant RP1 bearing cells regarding their adhesion properties were noted. In cell culture, stable expression of RP1 by itself led to a decrease of adhesion with the most pronounced effect in the RP1-ASP^236^ mutant cells. RP1-ASP^236^ containing HEK293 cells were more frequently found floating and were easier detachable from the underlying surface than empty vector- or RP1-wt containing HEK293 cells. Moreover, RP1-ASP^236^ and to a lower degree RP1-wt and RP1-ALA^236^ containing HEK293 cells were slower to re-attach after detachment compared to empty vector-containing HEK293 cells (Fig. S1).

**Figure 2:**
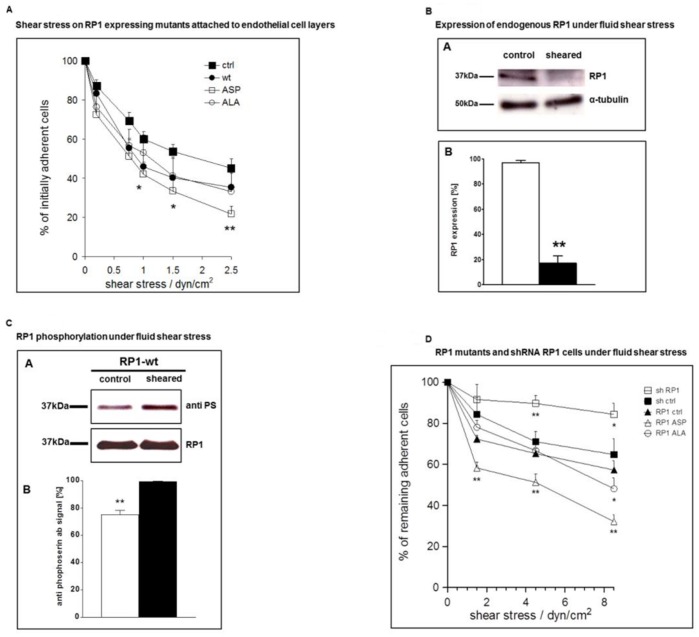
Shear Stress experiments. 2A Analysis of shear stress-dependent adhesion of RP1 mutants on endothelial cells under flow 1×10^5^ HEK293 cells stably transfected with different RP1 mutants were allowed to settle for 3 min on parallel plate flow chambers with pre grown confluent HUVECs. Subsequently, preheated HBSS/0.1% BSA was flushed through the chambers at the indicated calculated shear stress, and shear stress levels were increased every 30 s. Photographs were taken and adherent cells were counted in four fields for every condition. Cell line with empty vector (black squares), RP1 wild type (wt) (black circles), RP1-ALA^236^ (ALA) mutant (white circles), RP1-ASP^236^ (ASP) mutant (white squares). Values are means of n = 5–6+/− SEM. Asterisks denote statistically significant differences *p<0.05 or **p<0.01 between parental cell line and ASP mutant as determined by a two-tailed t-test. **2B Analysis of RP1 expression under fluid shear stress** Native HEK293 cells were exposed to fluid shear stress or simply cultured (control). Thereafter, cells were lysed and total protein from the lysates was employed in immunoprecipitation of RP1. Endogenous RP1 was detected by an RP1 specific antibody. α-tubulin served as a loading control. RP-1protein detected by Western blot was quantified using the ImageJ software. Asterisks mark statistically significant differences **p<0.01 between sheared and non-sheared cells as determined by a two-tailed t-test. **2C** **Analysis of RP1 phosphorylation under fluid shear stress** HEK293 cells overexpressing RP1-wt were exposed to 1dynes/cm^2^ shear stress. From cell lysate RP1 was immunoprecipitated and subjected to Western blotting. In parallel the phosphorylation status of RP1 was detected with an anti phospho-serine antibody (anti PS). As control HEK293 cells overexpressing RP1-wt were cultured without shear stress and otherwise processed alike. Total RP1 (RP1) expression served as loading control. The difference between phosphorylation intensity in sheared versus control cells was statistically significant (**, p<0.05). **2D Analysis of RP1 shRNA regulated cells and RP1 phosphor-mutants under fluid shear stress** HEK293 containing empty vector or various mutants were exposed to increasing shear stress. The curves show the percentage of adhering cells under different shear stress intensity (0, 1.5, 4.5 and 8.5 dynes/cm2) on control cells transfected with irrelevant shRNA served as a reference (black boxes) and were compared to RP1 specific shRNA bearing cells and the mutant cell lines RP1-ALA236, RP1-ASP236. A significant gain of cell adhesion is seen for RP1 specific shRNAs (white boxes). The statistical differences of adherent cells between the depicted cell lines and the control cells are marked with Asterisks *p<0.05 or **p<0.01.

To quantify the observed changes in cell adherence a shear stress assay was performed. Adhesion of HEK293 cells stably transfected with empty vector (control) or RP1-wt or RP1-ALA236 or RP1-ASP236 on a surface of endothelial cells (HUVEC) in parallel plate flow chambers at defined shear stresses were assessed. Thereby, the influence of RP1 function on resistance of HEK293 cells to detachment upon increasing levels of shear stress as a measure of the adhesion function could be evaluated. With increasing shear stress adhesion of HEK293 cells was declining to 20–45% at 2.5 dyn/cm2. The differences in adhesion behavior under flow between the parental cell line, RP1-wt or the RP1-ALA236 mutant did not reach statistical significance. In contrast, the RP1-ASP236 bearing cells displayed significantly less adhesion from 1 to 2.5 dyn/cm2 compared to the control cell lines (Fig. 2A). This indicates that expression of RP1-ASP236 leads to decreased cell adhesion under static and flow conditions.

To corroborate these findings, we investigated the cellular amount of endogenous RP1 under shear stress conditions. After applying shear stress of 1 dynes/cm2 on native HEK293 cells over five hours we found endogenous RP1 expression to be reduced to almost undetectable levels by Western blotting (Figure 2B).

Next, we examined the RP1 phosphorylation status under fluid shear stress. For this, the RP1-wt containing mutant clone was chosen, because endogenous RP1 had been diminished after shear stress as described above (Fig. 2B) and would not be accessible for serine phosphorylation status examination. Hence, RP1-wt overexpressing cells were put under shear stress and the respective phosphorylation status was subsequently checked. After immunoprecipitation of RP1 from whole cell lysates serine phosphorylation of RP1 was revealed by blotting with an anti-phosphoserin antibody. RP1-wt bearing cells displayed a more than 20% increased serine phosphorylation of RP-1 after shear stress as seen in Figure 2C. The difference was statistically significant.

Finally, we performed the shear stress procedure with different intensity (0, 1.5, 4.5 and 8.5 dynes/cm2) on HEK293 empty vector and the mutant HEK293 cells. Here, we compared control cells transfected with irrelevant shRNA, RP1 specific shRNA bearing cells and the mutant cell lines RP1-ALA236, RP1-ASP236 (Fig. 2D). The results correlated with our findings in the parallel plate flow chambers on a surface of endothelial cells (Fig. 2A). Moreover, we demonstrated a significant gain of cell adhesion represented by higher resistibility to increasing shear stress in RP1 specific shRNA containing cells compared to controls. The difference of adherence between the specific RP1 shRNA cell line and its respective control reached statistical significance at 4.5 and 8.5 dynes/cm2. In accordance with this result, increased attachment of RP1 shRNA containing cells compared to control cells were observed after a brief attachment and wash off procedure (Figure S2).

**Figure 3:**
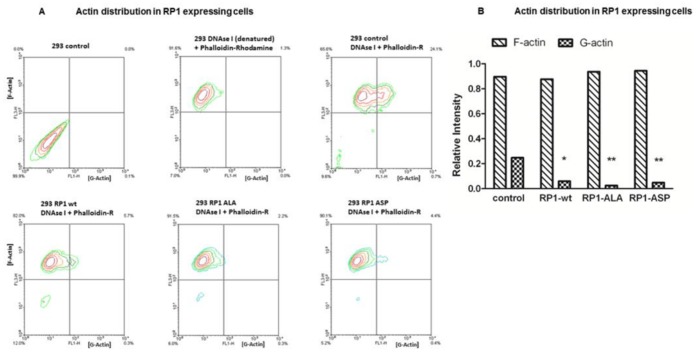
G- and F-Actin in RP1-expressing cells. **3A** G- and F-actin content of 10×10^5^ constitutively RP1 expressing cells were measured by FACS analysis. G-Actin (green) was measured in the FL-1 channel (Fluor 488, green) and F-Actin in the FL-3 channel (phalloidin rodamine staining, red). The upper left quadrant of each panel represents the F-actin pool, the upper right quadrant the G-Actin pool. The top three panels are the controls: Upper left panel, unstained control cells. Upper middle panel: Boiled fluoresceine conjugated DNAse I unable to bind G-Actin serving as a negative control. Upper right panel: Double staining of HEK293 cells with Fluor 488 conjugated DNAse I and Phalloidin-Rhodamine carrying the empty pEAK8 vector to determine the general content of G-actin and F-actin pools as reference. The bottom panels show the respective degree of G-actin decrease seen in the RP1-wt, ALA, ASP containing cell lines. **3B** Quantification of G-Actin content in RP1 expressing HEK293 cells compared to mock transfected cells from **3A**. Differences marked by asterisks were statistically significant using the two-tailed Fisher’s exact test (**p = 0.0001;*p = 0.0003).

To understand the underlying changes we examined the cytoskeletal properties of these cell lines in more detail. Here, the actin cytoskeleton of our RP1 clones displayed a significant difference with regard to G-actin amounts (Fig. 3A and 3B). A lower G-actin content in all RP1 expressing cell lines was detectable by flow cytometry. HEK293 control cells expressing the empty vector (Fig. 3A upper right panel) had a G-actin content of 24.1%. In RP1-wt, RP1-ALA^236^ (ALA), RP1-ASP^236^ (ASP) cells significantly reduced G-Actin contents were found (5.7% -wt, 2.2% ALA, 4,4% ASP, Fig. 3A, lower panels from left to right). In this regard Phospho- and non-Phospho-mimicking mutants were not different compared to RP1 wild type expressing cells.

**Figure 4:**
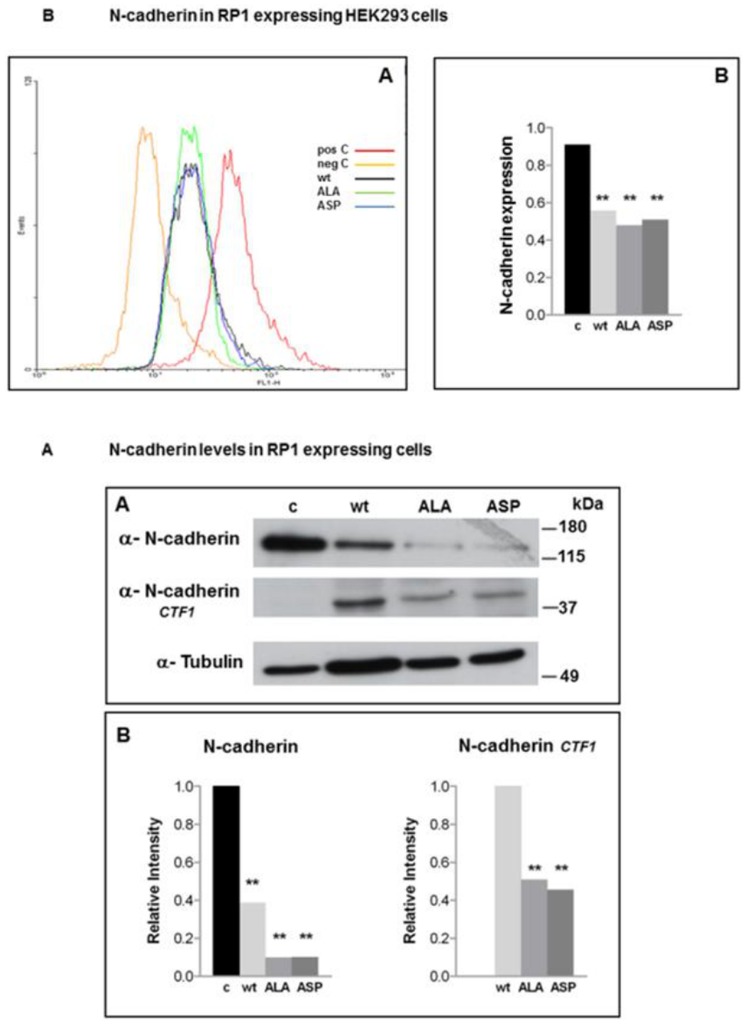
Cadherin expression in RP1-mutants. **4A** N-cadherin levels of HEK293 cells containing empty vector (c), wt, ALA and ASP were determined by immunoblotting with an N-terminal N-cadherin antibody. The middle panel shows a processed N-cadherin fragment (named CTF1) detected by a fragment specific antibody in respective lysates. The lower panel shows the β-tubulin loading control. **4B** The Western blot signals of **4A** were quantified using the Image-J software (Rasband, W.S., ImageJ, U. S. National Institutes of Health, Bethesda, Maryland, USA, http://rsb.info.nih.gov/ij/, 1997–2008). Differences marked by asterisks were statistically significant using the two-tailed Fisher’s exact test (**p<0.001) comparing control versus wt and mutants regarding complete N-cadherin and comparing wt versus mutants regarding N-cadherin cleavage fragment (CTF1). In the right panel, the empty c lane indicates no detectable CTF in control cells. **4C** N-cadherin levels were measured by incubation with a monoclonal antibody directed against the cytoplasmic tail and subsequent FACS analysis. The negative control (yellow line) was incubated with secondary antibody only. The positive control (red) was empty vector containing HEK293 cells. The results for HEK293 expressing RP1-wt are depicted in black, RP1-ALA^236^ in green and RP1-ASP^236^ in blue. **4D** Quantification of N-cadherin levels from FACS analysis**.** Differences marked by asterisks were statistically significant using the two-tailed Fisher’s exact test (**p<0.001).

These observations led us to investigate the surface levels of various adhesion molecules. When RP1 clones were tested for expression of Cadherins (N-, E-, P-) by Western blotting, a marked decrease of N-cadherin in cells expressing RP1 constructs compared to parental or empty vector-transfected cells (Fig. 4A) was noted. The difference of total N-cadherin levels among the RP1 clones; namely the RP1-wt, RP1-ALA^236^ or RP1-ASP^236^ mutant were not statistically significant (Fig. 4B left panel). Further, more cleaved N-cadherin (Fig. 4A middle blot) was found in the RP1 containing cells, with the significant highest amount of the N-cadherin fragment CFT1 in the RP1-wt clone (Figure 4B). This result hints towards a N-cadherin regulation by degradation. To reconfirm these results in a second assay, a FACS analysis utilizing an antibody against N-cadherin was performed (Fig. 4C). The mean fluorescence intensity (MFI) of cells containing RP1-wt, -ALA or -ASP was more than 2-fold lower than the empty vector. No significant differences among the various mutants were seen, neither in FACS analysis nor in immunofluorescent staining (data not shown).

## Discussion

Previously, we have found RP1 mRNA to be up-regulated upon T lymphocyte activation [4]. Since then it has become clear that RP1 (EB2) has distinct properties within the EB1 protein family despite its high homology to the other family members. However, more detailed cellular regulatory mechanisms of this EB1 family protein have remained elusive. New evidence indicates an important role of RP1 in malignancy [10], thus functional studies on RP1 are warranted. Here we report that RP1 can be phosphorylated by CK2 and the potential consequences of this interaction. Protein-protein interaction between the kinase and its target has been confirmed by co-immunoprecipitation. Our *in vitro* assays map the CK2 phosphorylation site of RP1 to serine 236 (Fig. 1).

CK2 is an ubiquitous kinase constitutively active with autophosphorylating properties [22]. Abundant evidence suggests that CK2 supports a cancer phenotype by influencing a number of cellular events including proliferation, anti-apoptosis and adherence [15], [23]. Though CK2 is often found overexpressed and deregulated in cancer [14], [24] it has never been found mutated. Its part in cancer development is not transforming cells but appears to be supporting a malign phenotype by protecting cancer cells against cellular defense programs activated by stress [25], [26]. For this CK2 is regarded a prototypical “non-oncogene target”. Inhibitors for clinical purposes have been developed [27] and the kinase is regarded as a novel key target in oncological therapy.

After having confirmed RP1 as the genuine CK2 target we created various mutants, to address consequential effects of RP1 phosphorylation on cellular phenomenology. Recent findings have indicated an involvement of RP1 in highly nerve invasive pancreatic cancer cells [10]. Pancreatic cancer cells examined by immunofluorescence have a specific cellular pattern for RP1 and actin. In cells expressing low levels of RP1 actin is distributed apical and accumulates in filopodia structures. In cells with high RP1 levels cortical and transverse stress fibers are found and RP1 colocalizes with abundant filamentous actin [10]. We find a decrease of G-Actin in RP1 containing clones (Fig. 2B) and suggest that RP1 is more involved in actin organization than hitherto assumed. In this context it is noteworthy that RP1 possesses an actin binding element located within its N-terminal domain and in contrast to EB1 and EB3, RP1 has significantly less microtubulin binding and bundling properties [5]. Besides actin changes constitutive RP1 expression results in downregulation of N-cadherin. Low levels of N-Catherine has been associated with less cellular adhesion [28] and when N-cadherin is cleaved by ADAM10 cell migration is promoted in glioblastoma cells [28]. In our model Phospho-mimicking and non-Phospho-mimicking did not differ significantly with regard to downregulation of N-cadherin, indicating no clear association between RP1 phosphorylation status and N-cadherin levels. However, in the functional assay the phospho-mimicking mutant RP1 ASP^236^ had the most profound phenotype as reflected by the least resistance to shear stress. Expanding on RP1’s role in adhesion, we found that shear stress leads to increased RP1phosphorylation. Furthermore, endogenous RP1 protein is downregulated in cells under shear stress and RP1 shRNA knockdown results in markedly increased adhesion. Taken together, these data support the notion that after an initial phosphorylation event of RP1, the protein is degraded and cellular adhesion is consecutively increased.

In summary, our data show that RP1 is a genuine phosphorylation target of CK2 and support a role of RP1 as a switch for CK2 in increasing cell adhesion properties. CK2 inhibitors are on the brink of clinical use but inhibition of CK2′s broad spectrum may be accompanied by unexpected side effects. By defining CK2 downstream targets like RP1 a more targeted approach may ultimately be developed if the present CK2 kinase inhibitors should not live up to their promises.

## Supporting Information

Figure S1 Surface attachment of RP1-wt and mutant RP1 expressing cells. Pictures show decreased attachment behavior of RP1-ASP236 mutant. Equal quantities 5×106 Cells containing empty vector (control left panels), RP1-wt (middle panels) and RP1 ASP (right panels) were seeded to 10 cm dishes and left for 30 minutes before supernatant and unsettled cells were washed off. 24 hours (upper panels) and 48 hours (lower panels) later, representative overview pictures of the respective cells were taken.
(TIF)

Figure S2 Surface attachment of of RP1 shRNA expressing cells. Pictures show increased attachment behavior of RP1 shRNA regulated cells (right panel) compared to control shRNA containing cells. Cells were seeded and washed off as described in S1. Overview pictures were taken at indicated time points.
(TIF)

Figure S3 RP1 shRNA Downregulation of endogenous RP1 protein in HEK293 cells by a specific lentiviral transduced shRNA (right lane) and a respective control shRNA (left lane) is shown by Western blotting. α-tubulin served as a loading control.
(TIF)
