# Supplementary figures and images for: RP1 Is a Phosphorylation Target of CK2 and Is Involved in Cell Adhesion
Source: PLoS One. 2013 Jul 3;8(7):e67595. doi: 10.1371/journal.pone.0067595 (PMC3701075; doi:10.1371/journal.pone.0067595)

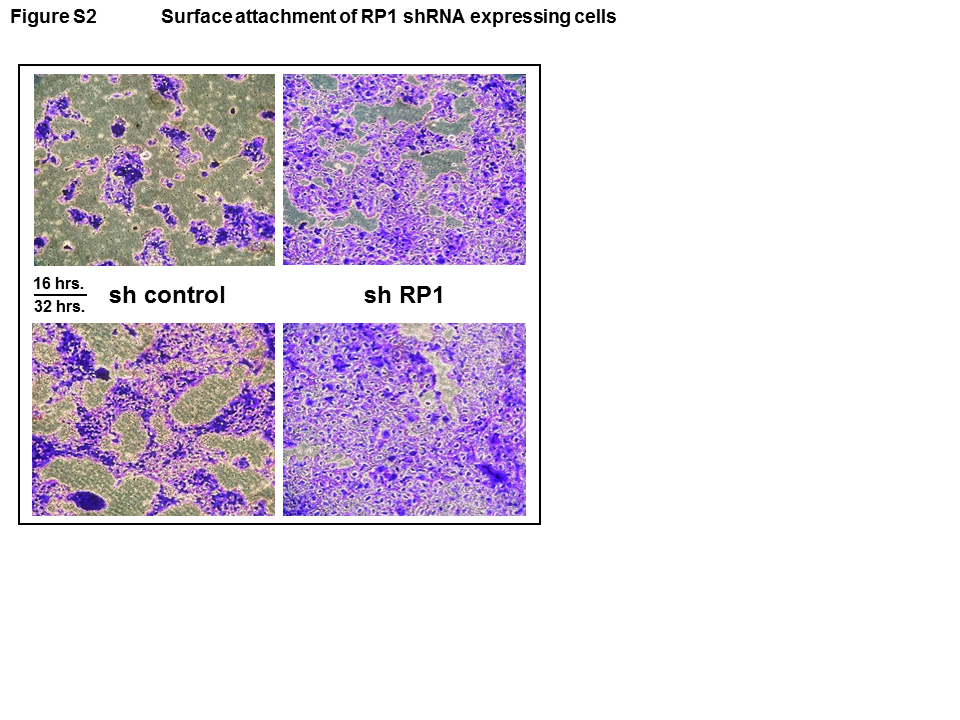

Supplement: Figure S2 — Surface attachment of of RP1 shRNA expressing cells. Pictures show increased attachment behavior of RP1 shRNA regulated cells (right panel) compared to control shRNA containing cells. Cells were seeded and washed off as described in S1. Overview pictures were taken at indicated time points. (TIF) [file pone.0067595.s002.tif]

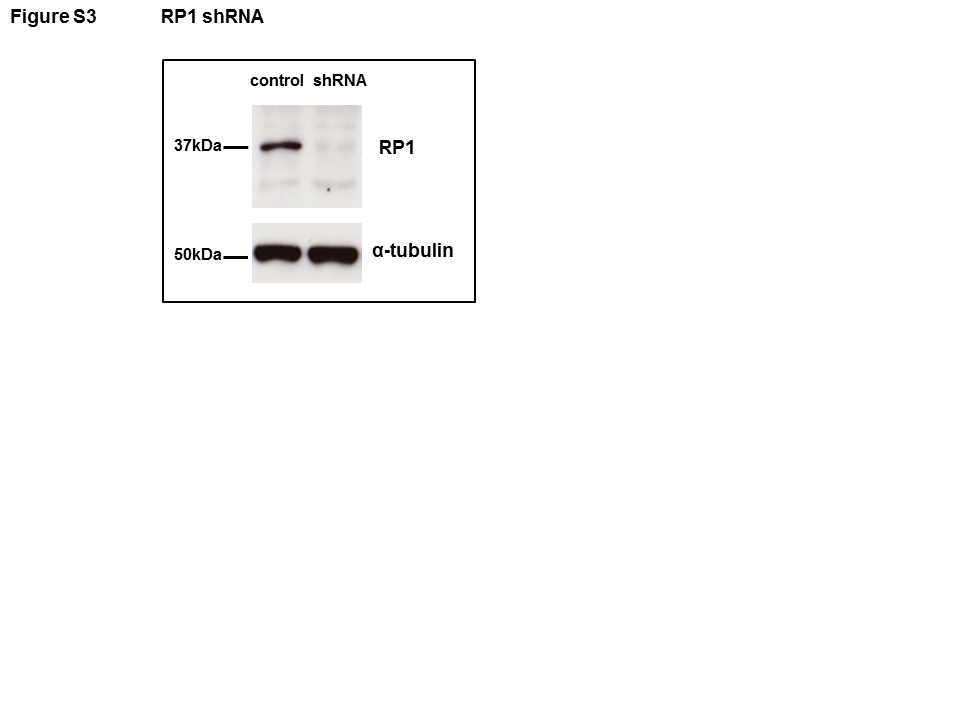

Supplement: Figure S3 — RP1 shRNA Downregulation of endogenous RP1 protein in HEK293 cells by a specific lentiviral transduced shRNA (right lane) and a respective control shRNA (left lane) is shown by Western blotting. α-tubulin served as a loading control. (TIF) [file pone.0067595.s003.tif]
